# The impact of COVID‐19 on patient engagement in the health system: Results from a Pan‐Canadian survey of patient, family and caregiver partners

**DOI:** 10.1111/hex.13421

**Published:** 2022-01-13

**Authors:** Laura Tripp, Meredith Vanstone, Carolyn Canfield, Myles Leslie, Mary Anne Levasseur, Janelle Panday, Paula Rowland, Geoff Wilson, Jeonghwa You, Julia Abelson

**Affiliations:** ^1^ Department of Health Research Methods, Evidence and Impact McMaster University Hamilton Ontario Canada; ^2^ Department of Family Medicine McMaster University Hamilton Ontario Canada; ^3^ Centre for Health Economics and Policy Analysis (CHEPA) McMaster University Hamilton Ontario Canada; ^4^ Patient Advisors Network (PAN) Canada; ^5^ Department of Family Practice University of British Columbia Vancouver British Columbia Canada; ^6^ School of Public Policy University of Calgary Calgary Alberta Canada; ^7^ Department of Community Health Sciences, Cumming School of Medicine University of Calgary Calgary Alberta Canada; ^8^ Department of Occupational Science and Occupational Therapy, Temetry Faculty of Medicine University of Toronto Toronto Ontario Canada; ^9^ The Wilson Centre University of Toronto/University Health Network Toronto Ontario Canada; ^10^ Nova Scotia Health Authority Halifax Nova Scotia Canada

**Keywords:** COVID‐19, patient engagement, patient involvement

## Abstract

**Introduction:**

The COVID‐19 pandemic has had an impact on all aspects of the health system. Little is known about how the activities and experiences of patient, family and caregiver partners, as a large group across a variety of settings within the health system, changed due to the substantial health system shifts catalysed by the pandemic. This paper reports on the results of a survey that included questions about this topic.

**Methods:**

Canadian patient, family and caregiver partners were invited to participate in an online anonymous survey in the Fall of 2020. A virtual snowballing approach to recruitment was used. Survey invitations were shared on social media and emailed to health system and governmental organizations with the request that they share the survey with patient partners. This paper focuses on responses to two questions related to patient partner experiences during the COVID‐19 pandemic.

**Results:**

The COVID‐19 questions were completed by 533 respondents. Over three quarters of respondents (77.9%, *n* = 415) indicated their patient engagement activities had been impacted by COVID‐19. The majority (62.5%, *n* = 230) experienced at least a temporary or partial reduction in their patient engagement activities. Some respondents did see increases in their patient engagement activities (11.4%, *n* = 42). Many respondents provided insights into their experience with virtual platforms for engagement (*n* = 194), most expressed negative or mixed experiences with this shift.

**Conclusions:**

This study provides a snapshot of Canadian patient, family and caregiver partners' perspectives on the impact of COVID‐19 on their engagement activities. Understanding how engagement unfolded during a crisis is critical for our future planning if patient engagement is to be fully integrated into the health system. Identifying how patient partners were engaged and not engaged during this time period, as well as the benefits and challenges of virtual engagement opportunities, offers instructive lessons for sustaining patient engagement, including the supports needed to engage with a more diverse set of patient, family and caregiver partners.

**Patient Contribution:**

Patient partners were important members of the Canadian Patient Partner Study research team. They were engaged from the outset, participating in all stages of the research project. Additional patient partners were engaged to develop and pilot test the survey, and all survey respondents were patient, family or caregiver partners. The manuscript is coauthored by two patient partners.

## 1 INTRODUCTION

The onset of the COVID‐19 pandemic required health care systems to make significant changes very quickly to address the health and patient care needs associated with this public health crisis. All individuals working in health care systems have been impacted by these changes, including patient, family and caregiver partners who are engaged in a wide range of activities across the health system through advisory and leadership roles in governance, policy, planning and service design. The ethical and experiential aspects of the pandemic response, such as the prioritization of health care services,^1^, ^2^ the allocation of vaccines and triaging of life‐saving treatment^3^, ^4^, ^5^ and the development of visitor restriction policies,^6^, ^7^ suggest numerous opportunities to incorporate patient perspectives into health policy, planning and delivery activities. Despite this context, however, it is unclear how patient, family and caregiver partners were engaged in the system during COVID‐19, and how they experienced engagement during the pandemic.

Initial reports from around the world about patient engagement activities during COVID‐19 show many challenges. In France, patient partners were not included on expert committees or consulted regarding lockdown measures,^8^ and in the United Kingdom, there was a dramatic decrease in the number of research funding applications that incorporated patient engagement during the pandemic.^9^ Work out of Australia suggests that consumer engagement activities occurred less frequently. When they did occur, activities focused mainly on COVID‐19 with other important work being cancelled. Further, the switch to virtual platforms tended to disadvantage those who had low technological literacy or income.^10^ In Ontario, Canada, patient engagement within the newly established Ontario Health Teams paused or diminished during the first wave of the pandemic due to competing priorities of member organizations.^11^ Additional concerns remain regarding specific groups, such as people with disabilities, who have been left out of discussions regarding COVID‐19.^12^

Other examples highlight how patient engagement has continued during the COVID‐19 pandemic. One Canadian health delivery organization, for example, implemented several strategies, including monthly town halls and forums in addition to their existing Patient and Family Advisory Committee meetings, to ensure patients and families were engaged throughout the pandemic.^13^ Similarly, an American health organization reported on how they successfully pivoted to virtual meetings to continue the work of Patient and Family Advisory Councils (PFACs).^14^

Many of these reports are single case studies that focus on a small number of personal experiences. What is unknown at this stage is how the activities and experiences of patient, family and caregiver partners, as a large group across a variety of settings, have changed due to the substantial health system shifts catalysed by the pandemic. This paper reports on the results of the Canadian Patient Partner Study (CPPS) survey conducted in Fall 2020 that included questions about this topic.

## 2 METHODS

The CPPS survey, an anonymous internet‐based survey, collected responses from October to December 2020, corresponding to the second major wave of COVID‐19 in Canada. Eligibility for the survey included persons who had ever participated in activities with a Canadian health system or governmental organization with the aim of building on their lived experiences as a patient, family member or informal caregiver to inform the organization's governance or evolution. While we refer to these individuals as ‘patient partners’, caregivers and family members are included in this group. This was explicitly stated at the beginning of the survey. The survey drew on an extensive review of the patient partner literature (completed during a previous phase of research), existing surveys in this field and in‐depth consultation and collaboration with patient partners from the Patient Advisors Network (PAN), two of whom are members of the research team. The survey was pilot‐tested and revised with input from patient partners in British Columbia, Ontario, Quebec and Nova Scotia.

The survey focused on patient partner experiences, attitudes and demographics and was available in French and English. While other patient partner surveys have been conducted locally,^15^ to our knowledge, this is the first survey of its kind to attempt to reach patient partners from across Canada in both official languages.

### 2.1 Sample

As there is no known list of patient partners in Canada, our sampling frame was unknown at the outset of the survey. Recruitment relied on a virtual snowballing approach.^16^ Members of the study team and the project's external advisory committee, comprising patient partners, engagement researchers and health system professionals, were asked to disseminate the survey invitation widely through their networks. The research team sent additional recruitment emails to health system and governmental organizations across Canada asking them to share the survey information directly with their patient partners. The survey was actively promoted on various social media platforms (Twitter, Facebook, LinkedIn) by various members of the research team at multiple points during the recruitment period. Social media posts encouraged followers to share the information through their networks. All respondents received an information letter, provided consent to participate and had an opportunity to enter a draw to win one of three $200 cash prizes. The Hamilton Integrated Research Ethics Board (HIREB) reviewed and approved this study (#10705).

### 2.2 Analysis

For this paper, the descriptive analysis focuses on responses to two survey questions related to patient partner experiences during the COVID‐19 pandemic. All respondents were invited to answer the question ‘Has the COVID‐19 pandemic had an impact on your patient partner activities?’. Those who responded ‘yes’ to this question were asked the open‐ended question, ‘Please describe how your patient partner activities have been influenced by the pandemic’. Frequencies were calculated for the initial COVID‐19 question (yes/no). *χ* ^2^ tests of independence were performed to examine the relationship between the impact of COVID‐19 on engagement activities and the demographic variables presented in Table 1.

We analysed the open‐ended comments using a two‐stage approach. First, we engaged in unconstrained deductive analysis^17^ to determine the impact of COVID‐19 on patient engagement. Previously defined categories (‘stopped’ and ‘continued’) were assigned to the open‐ended responses to the second question based on how respondents described the impact of COVID‐19 on their partnering activities. Just over half of the responses fit into these two categories. Our unconstrained approach was used to organize the rest of the responses into an ‘other’ category, which was then inductively analysed to provide further description. Within this category of responses, we used an inductive approach to conventional content analysis to organize the remaining responses (e.g., stopped then restarted, increased). Initial coding was completed by one coder (J. Y.) and then reviewed and confirmed by a second coder (L. T.). Each response category was further analysed by one coder (L. T.) to elaborate on the data within each category, specifically related to the impact of virtual engagement on respondents.^18^ French responses were translated into English for analysis purposes.

## 3 RESULTS

The question regarding the impact of COVID‐19 on patient engagement activities was completed by 533 respondents. Over three quarters of respondents (77.9%, *n* = 415/533) indicated that their work had been impacted by COVID‐19 in some way (Table 1). Forty‐one of these respondents completed the survey in French. The demographic characteristics of respondents are highlighted in Table 1. Respondents in both the impacted and not impacted by COVID‐19 groups were mainly female (76.1%, 74.6%), had a college or university education (84.6%, 89.8%) and White (85.5%, 83.1%). Respondents who reported their activities were not impacted by COVID‐19 were significantly more likely to indicate that they were in good or excellent health (*p* = .044, *p* < .05). No other demographic or geographic characteristics were statistically significant between those whose activities were impacted by COVID‐19 and those who were not (Table 1).

**Table 1:** Respondent characteristics (*n* = 533)

Characteristic	Impacted by COVID‐19 (*n* = 415)	Not impacted by COVID‐19 (*n* = 118)	*p*‐Value
Age (mean)	57.1 years	58.4 years	–
Province, % (*N*)			
New Brunswick, Newfoundland and Labrador, Nova Scotia, Prince Edward Island	12.5% (52)	11.9% (14)	.85
Quebec	11.8% (49)	13.6% (16)	.61
Ontario	36.9% (153)	37.3% (44)	.93
Alberta, Manitoba, Saskatchewan	19.5% (81)	16.1% (19)	.40
British Columbia and Northwest territories	15.4% (64)	17.8% (21)	.53
No response	3.9% (16)	3.4% (4)	–
Gender			
Male	20.2% (84)	21.2% (25)	.82
Female	76.1% (316)	74.6% (88)	.73
Transgender	0.7% (3)	0.8% (1)	.89
Nonbinary	0.002% (1)	0% (0)	–
Prefer not to answer/no response	2.7% (11)	3.4% (4)	–
Highest level of education			
College or university	84.6% (351)	89.8% (106)	.15
Health status			
Very good or excellent	36.4% (151)	46.6% (55)	.044**
Race category			
White	85.5% (355)	83.1% (98)	.50

**Significant at *p* < 0.05.

### 3.1 Overall experiences with engagement during COVID‐19

Respondents who indicated their engagement activities had been impacted by COVID‐19 were asked to respond to the open‐ended question ‘please describe how your patient partner activities have been influenced by the pandemic’. In response to this question, 368/415 respondents (88.7%) provided insights into how the pandemic had influenced the level of their patient engagement activities, specifically commenting on if their activities had remained consistent, decreased, stopped or increased during the first and second Canadian waves of the COVID‐19 crisis (from March 2020 to Fall 2020) (Table 2).

**Table 2:** Impact of COVID‐19 on patient engagement activities (*N* = 368/415533)

Type of impact	% (*N*)	Illustrative quote
Partnering activities remained consistent^a^	26.1% (96)	‘I still am involved in lots of engagements. Pre‐COVID, I liked to show up in person to do meeting but now it is all over zoom’
Partnering decreased	26.9% (99)	‘Most activities on hold. I'm not doing much as a patient advisor at my hospital. However, some of my activities with other organizations continue because by necessity meetings were always held on Zoom as participants live in different cities’
Partnering decreased due to personal factors	4.3% (16)	‘Lock‐downs prompted a widespread work‐from‐home situation and without that happening, I wouldn't be employed right now, and my work hours have definitely interfered with participating as the meetings are during my work hours’
Partnering stopped	24.2% (89)	‘There was dead silence for months…committee stopped meeting. When I reached out to provide the patient voice, I either had the door slammed in my face or silence. COVID stopped all patient engagement—at a time they truly needed to hear from patients!!!’
Partnering stopped, then restarted	7.1% (26)	‘There were no committee meetings for a time, now the meetings are on zoom’
Partnering activities increased	11.4% (42)	‘My role, and that of my fellow board members has been intensified and altered by the pandemic. There were new issues that arose plus the change in meeting formats’

^a^ Individuals who indicated that their activities were not impacted by COVID‐19 did not reply to this question. It could be assumed that these 118 individuals' patient engagement activities remained consistent, as well. As a result, this number may be underreported.

Through their description of the impacts of COVID‐19 on their engagement work, more than half of respondents described experiencing at least a temporary decrease in the amount of patient partnering work they were involved with during the pandemic (62.5%, *n* = 230). This included describing decreases in the number of activities they were involved in or the frequency of these activities due to changes made by organizations (26.9%, *n* = 99) or personal situations (4.3%, *n* = 16). A small number of respondents described how their patient engagement work stopped and then resumed later in the pandemic (7.1%, *n* = 26), and just under a quarter of respondents described how their activities stopped and had not yet resumed (24.2%, *n* = 89). Others described how their engagement work remained relatively constant despite the need to move to virtual platforms and other modes of working (26.1%, *n* = 96) and a small number (11.4%, *n* = 42) reported an increase in their partnership activities during the pandemic.

#### 3.1.1 Decreased patient engagement activities

Over half (62.5%, *n* = 230) of the respondents who commented on how their engagement activities were impacted during the COVID‐19 crisis described at least a temporary decrease in activity levels. Respondents' comments highlighted several reasons why engagement activities decreased or stopped. Some spoke to the shifting priorities of the institution to COVID‐19 response efforts as a reason: ‘the healthcare system has had to focus on COVID‐19 which means that a lot of other programs have been put on the backburner’. Others highlighted the difficulty in transitioning certain engagement activities to telephone or virtual platforms as a reason for the pause. In other cases, committees and groups continued but patient partners were not invited to participate due to a lack of access to the required technology (e.g., laptops to join zoom meetings; Wi‐Fi) or an inability of the organization to provide the individuals with access (e.g., non‐employees could not access the required platforms/networks).

While respondents' comments suggested they understood why this decrease or stop in activities happened, some expressed frustration with how it occurred. Challenges included a lack of communication, uncertainty around next steps and a sense of being cut out of the work: ‘There was a time period early in the pandemic when all engagements basically shut down which was understandable. But communication to patient partners as to when they might be running again, etc. was not very good’. Respondents who commented about being left out or cut out of the work expressed strong reactions to this. A small group of respondents expressed how they felt abandoned or ignored by the organizations and groups they partnered with. Some described this as a ‘great shock’ given the length and depth of their past relationships and felt as if groups had ‘tossed us overboard like extra baggage’.

A major category in the responses was the frustration about not engaging patient partners at a time when decisions were made that would directly impact fellow patients and caregivers, despite involvement in these types of decisions pre‐COVID‐19. Respondents noted they had been left out of decision‐making at a time when patient voices could be most critical: ‘we were dropped like a hot potato during the pandemic. It was like showing up for work to find the doors locked. No one reached out, decisions were being made that affect me as a patient, but I wasn't included in the discussion’. Some respondents were not told why they were excluded, while others were told it was too time‐consuming to include patient, family and caregiver perspectives during a crisis: ‘I am being left out of pertinent decisions as researchers have said they don't have time to consult patient partners on decisions because those decisions need to be made rapidly’. This sudden lack of inclusion in the work of the health system left some respondents questioning if their contributions were truly ever valued: ‘After March 15 I had empty days in my calendar…that told me that all the effort I had put in to make myself visible, helpful and part of the team, was not valued or deemed necessary…Very disappointing, if my ideas had been heard, perhaps less people would have died or we would have less mental health issues’.

For most respondents, it was organizations that altered patient engagement activities because of rapid refocusing on COVID‐19. For a small number of patient partners, however, the change in their engagement activities was a result of personal obligations such as caregiving activities or due to issues with technology that made participation during the pandemic difficult or impossible (4.3%, *n* = 16). One respondent summarized by saying they were unable to participate in virtual engagement activities as ‘I wasn't sure how to use the particular software, and I was exhausted from all the additional COVID‐19 related things—homeschooling children, online grocery ordering and other duties took priority’. Other reasons cited for stepping back from patient engagement work at this time included conflicts with their work schedule, increased caregiving responsibilities due to the lack of support during the pandemic, lack of access to Wi‐Fi, computers and other technology, and the need and desire to quarantine and reduce exposure to others during this time.

#### 3.1.2 Increase in patient engagement activities

While many respondents reported that their activities lessened or stopped during this time, 11.4% (*n* = 42) reported that their patient partnering activities increased during the COVID‐19 pandemic. A few respondents spoke of how their engagement work seemed busier due to more meetings or changes in activities as their ‘involvement has increased and [they] have more tasks’. Some had more time available to participate due to changes such as working from home or participating in engagement activities virtually: ‘I am involved in many more activities than prior to the pandemic because the rest of my life has stopped and I have more time, plus everything is done virtually’.

Other respondents identified COVID‐19 itself as the reason for increased engagement as they became engaged in COVID‐19‐related projects and research studies and in efforts related to testing and supporting the pandemic response. Some respondents also identified the need to participate in advocacy efforts due to the impacts of COVID‐19 on patients, families and caregivers. In contrast to those respondents who felt like their organizations dropped patient engagement completely, some respondents identified that their partner organizations relied more heavily on patient partners during this time: ‘My health authority has called upon patient partners more frequently during the pandemic than it did previously. It is now consulting us on a wide variety of topics irrespective of the particular committees we sit on’. Not only were some patient partners engaged more frequently during the pandemic, but it was noted that due to the speed at which things changed during this time, it was easier for some to see the impacts of their contributions on the organization: ‘There is almost daily consultation with advisors and because change is happening so rapidly, we get to see how our input has influenced organizational decision making’.

### 3.2 Experiences with virtual platforms for patient engagement

While the survey did not prompt respondents to reflect on their experiences with virtual platforms for patient engagement, just under half (46.7%, *n* = 194/415) of respondents commented on this aspect of their experience. Given the requirements to socially distance as well as reduce travel and contacts, most engagements that continued during the pandemic were held virtually or by telephone. Experiences with the virtual platforms varied, with many respondents identifying both positive and negative impacts. While some respondents highlighted only positive experiences with virtual engagement, most expressed negative experiences with this shift or identified that the shift to virtual led to both positive and negative or more neutral experiences (Table 3).

**Table 3:** Experiences with virtual platforms for patient engagement (*N* = 194/415)

Type of impact	% (*N*)	Illustrative quote
*Negative experiences*		
Interpersonal challenges	26.8% (52)	‘It has become less enjoyable. Part of the reason I became involved was to improve my social life. I had retired from a successful business career and participation in these programs has helped me as much as I have helped the projects. The pandemic has generally made participation less enjoyable due to limitation of social connection within virtual meetings’
Quality of engagement is reduced	17.0% (33)	‘Most engagement is done in video conferencing and sometimes it can be problematic. Harder to get your voice heard or be able to read the room to ensure others are supportive’
Technological barriers to engagement	13.4% (26)	‘IT glitches stress me out and affect my desire to participate. Hard to stay focused and committed online. Not impossible, just less than ideal. Our virtual platforms are way too varied’
Generally struggling with virtual	7.7% (15)	‘Most [activities] were cancelled then went virtual, which is hard’
Loss of compensation	2.1% (4)	‘Since the expense checks were, in fact, my only source of income, I lost it when things went virtual but am not eligible for any income replacement programs’
Too many virtual meetings	2.1% (4)	‘Another way the pandemic has affected me is that I actually am attending more meetings that I truly wish to attend. Meetings are increased (due to savings in travel time), but that means I have meetings conflicting at the same time. I am even at the point where I sometimes am attending and listening to two or three meetings at the same time even though everyone sees me online in just their meeting’
*Positive experiences*		
Virtual engagement is fine, made things easier	17.0% (23)	‘I'm actually able to better participate because I couldn't always make meetings but Zoom makes it much easier to be involved. In the past, when I would call into a meeting, it was very hard to participate and feel included…but now everyone is online, so that's a good thing’
Less travel, less wasted time	7.2% (14)	‘We are working remotely, which actually allows me to take on a lot more than I typically would be capable of’
Increased accessibility because of virtual engagement	6.7% (13)	‘I am involved in many more activities than prior to the pandemic because the rest of my life has stopped, and I have more time plus everything is done virtually. Due to chronic conditions travelling to downtown and other locations made it difficult to impossible to do so. It often was an expense to me because I had to take community drive program. Even where compensation was offered, it did not cover these costs’
Created new opportunities	5.2% (10)	‘It has definitely increased my ability to attend meetings and conferences as they are all being held virtually’

#### 3.2.1 Negative experiences

Interpersonal challenges (26.8%, *n* = 52) were the most mentioned negative experience associated with the move to virtual patient engagement activities. Respondents identified the lack of informal in‐person interaction during meetings (e.g., coffee breaks, lunches) as a significant loss that affected their functioning as a committee: ‘The ability to network with others… gone. Sometimes this crucial info exchange would happen before/during/outside of meetings we happened to be invited to. There are a lot of webinars, but no avenues for advocates to exchange info “off the record”, or as a group to discuss emerging issues, brainstorm, plan ahead’. The lack of interaction also had negative impacts on individuals' mental health. For many, patient engagement activities provide a social outlet and the move to virtual engagement activities changed this experience, compounding the already difficult social situation of the pandemic given the need for isolation and social distancing: ‘I am a senior and live alone…my volunteer activities were very important in my social contacts with colleagues and “workmates” which are now absent. I miss that aspect of my volunteer work so much’.

Some respondents who struggled with virtual engagement expressed that they felt that the quality of the engagement activities was reduced when undertaken virtually (17.0%, *n* = 33). For some, seeing others was a critical part of virtual engagement and when that was not possible, the quality was significantly decreased: ‘Zoom meetings have deteriorated into effectively being telephone meetings as people don't turn on their cameras so no chance to see the participants. I don't know if they are bored, not understanding, not paying attention, agreeing, wanting to say something, etc, etc’. More specifically, the quality of the engagement was reduced when some individuals participated in person (e.g., health system staff) while others (e.g., patient partners) participated virtually. This made it difficult for those not in the room to fully participate and engage in discussion. Generally, these individuals reported that the quality of the engagement was simply poorer: ‘For me, [Zoom meetings] constitutes an obstacle to achieving the objectives of my role as a patient partner. The in‐person contact can never be replaced by technology especially in regard to non‐verbal cues’. (Translated from French.)

A number of respondents commented how the switch to virtual was challenging due to technological barriers (13.4%, *n* = 26). These included needing to pay for internet access: ‘I am now paying for a wi‐fi service when I would normally use the library or the limited data on my phone plan to participate in some projects’ and dealing with issues related to meeting platforms, including how to use them, how to access them and the fact that they can be unreliable. As one respondent noted, ‘Everything is on Zoom and that in itself has presented many technical, engagement and collaborative challenges’.

Some respondents provided general comments about the struggles with virtual engagement (7.7%, *n* = 15) generally just struggling with virtual engagement—they spoke of how it ‘just is not the same’ and that ‘Zoom fatigue has set in’. A small number of respondents (2.1%, *n* = 4) commented on the lack of compensation and incentives for participation in virtual engagement activities—in some cases, this meant missing ‘those travel perks, which was a huge incentive’ or the ‘free meals’ that come with some in‐person meetings. In one case, the respondent spoke of how the compensation for engagement activities was their only source of income and virtual engagements meant this income source was lost: ‘Since the expense checks were, in fact, my only source of income, I lost it when things went virtual but am not eligible for any income replacement programs’.

#### 3.2.2 Positive experiences

For some respondents, the move to virtual engagement platforms was a positive change. It worked well and there were no concerns with it for most in this group (11.9%, *n* = 23). In some instances, it even facilitated their engagement activities. For example, one respondent noted that the organization they were working with found ways to engage them ‘with very specific questions in mind, sometimes via text message alone’, which they appreciated given the challenges of living through a pandemic, especially as a caregiver.

Increased accessibility was the other major positive impact a small group of respondents commented on (6.7%, *n* = 13). For patient, family and caregiver partners with caregiving responsibilities or health conditions that make travel or sitting in meetings difficult, or for those without easy or reliable access to transportation, virtual meetings made their patient engagement activities more easily accessible: ‘While this may sound counterintuitive, I have been able to participate in more patient partner activities during the pandemic as so much has been done on virtual platforms. I cannot sit long these days and would not have been able to travel to conferences or annual meetings during normal times…last year I had to back out of so many meetings due to pain’.

Some respondents (7.2%, *n* = 14) commented on virtual engagement as a way of saving time and increasing productivity as they do ‘not have to get to places physically and take all that transportation time on public transit’, allowing them, in some cases, to attend more meetings and spend more time on their engagement activities. Virtual engagement also increased the opportunities available to some patient partners (5.2%, *n* = 10): ‘the shift to virtual meetings has opened doors to new opportunities that I couldn't have taken on if the meetings were in person’. One individual noted that they took this time as an opportunity to ‘make more connections with patient partners across Canada….[and] redirected energy to how we can use the virtual platform to build our patient partner community’.

Many who saw advantages to virtual engagement, identified value in continuing the virtual approach after the pandemic, even if it was not their preferred way of participating: ‘I have noticed some people like the social distance and orderly structure of the Zoom [meetings]….it encourages them to speak up when perhaps they would not in person….I think this pandemic time will result in more technology being used and that will help caregivers and patients who find it difficult to attend in‐person meetings….having people participate both in‐person and using technology should be offered more often…hopefully both options will be more available in future’.

## 4 DISCUSSION

COVID‐19 has led to numerous changes within the health system and consequently has also led to many conversations about the impact of the pandemic on patient, family and caregiver engagement.^9^, ^10^, ^14^ There have been numerous anecdotal reports that patient, family and caregiver engagement largely stopped during the pandemic.^8^, ^19^ The results of this survey, however, suggest that while most Canadian patient, family and caregiver partners who participated were impacted by COVID‐19, a significant majority (over two thirds) continued their work even if there were delays or it was reduced.

Despite continued involvement in many ways, respondents to our survey expressed frustration that they were rarely included in COVID‐related committees and policy making when they felt that they could add significant value to these processes. Richards and Scowcroft highlighted the absence of those with lived experience in policy development during the outset of the pandemic and suggested that this absence reinforced the notion that patient, family and caregiver engagement, while embedded in health systems, is still seen as ‘“nice to have” but not essential’.^19^ Some of our survey respondents echoed this feeling highlighting that, despite the work that they had put in for years prior with organizations, the fact that they were so quickly dropped when the pandemic began was a sign that they were not as valued as they once thought. If organizations are truly embedding patient, family and caregiver engagement, the expectation is that it would and should continue during times of crisis. The fact that many respondents did report that their patient engagement activities continued, even if it was in an altered way or delayed, suggests that engagement remained a priority across several organizations. Further work to understand the differences between organizations where engagement continued, as compared to those where it stopped, could reveal how to support engagement within organizations at all times in spite of disruptions, in a truly embedded way.

It is acknowledged that many individuals who become patient, family and caregiver partners do so because of a negative interaction with the health system that they want to improve for others.^20^, ^21^ As a result, they may enter their patient partner roles with some mistrust in the health system, which their patient engagement activities may help to repair over time. The fact that nearly a quarter of patient, family and caregiver partners felt excluded during a time when they felt that their contributions were as, if not more, important than ever is especially problematic given this context. While many respondents acknowledged that the reduction in engagement was understandable as health systems were facing unprecedented challenges, communication around these changes was critical. The lack of communication combined with the sense of being left out challenges the rebuilding of trust. This lack of inclusion was also seen in other jurisdictions including the Netherlands, where Kleefstra and Leistikow identified how the exclusion of patient partners led to missed opportunities and highlighted how patient engagement was less embedded than once thought.^22^ As this crisis eventually subsides, planning for how to communicate and engage with patient, family and caregiver partners during future crisis situations and how to rebuild the trust lost will be critical.

As in other policy areas, the initial months of the pandemic have provided an opportunity to consider lessons for how engagement might improve in the post pandemic era. Given the wide range of policy responses to COVID‐19 across the world, including stay‐at‐home orders, social distancing requirements, closures of office spaces and restrictions on access to hospitals and other health settings, there has been a need to adjust ways of working across many fields, including patient, family and caregiver engagement. Respondents highlighted numerous ways in which the way that they did their work changed, specifically regarding the switch to virtual modes of engagement. Many respondents saw virtual engagement as being challenging, especially because of the access issues presented by technology and the loss of social contact. This echoes what has been seen in the literature, with technological barriers posing issues for patient engagement, both within engagement in direct care and in service improvements/recommendations.^23^ Respondents, however, also identified some important benefits. Virtual engagement allows individuals who find it difficult to travel to meetings, either due to their own health concerns, their caregiving responsibilities or the cost, to participate without leaving their homes. This may also make it easier for some individuals to participate in meetings during times when they would otherwise be unavailable. Further, respondents highlighted their ability to participate in engagement activities with organizations that were not local to them as an advantage, suggesting virtual engagement can lead to the ability to increase international collaboration. Other studies of patient engagement during COVID‐19 have highlighted the benefits of virtual collaboration, including increased participation and ensuring the patient voice remained at the front and centre during the pandemic.^14^

Providing access to patient, family and caregiver engagement activities in‐person and through virtual platforms may allow for individuals to participate in their preferred way, addressing both sets of needs. When this hybrid approach is taken, however, it is critical to ensure that those participating virtually are engaged equally to those who are in the room, as this was identified as a concern by some respondents. If there is a shift to virtual engagement in the longer term, there is also a need to support patient, family and caregiver partners' technological needs. Respondents spoke of the challenges in connecting to engagement activities virtually when they did not have access to Wi‐Fi, a good quality computer with a microphone and speaker, and other technological supports. This was particularly difficult for some, with the closure during the pandemic of facilities such as libraries where respondents may have had access to such supports. Providing supported technological access could help to ensure that all patient partners who want to participate virtually are able to do so, an important consideration to ensure equity and diversity.

This study is not without limitations. As there is no list of patient, family and caregiver partners in Canada, we do not know how the group we recruited compares to the overall population of Canadian patient, family and caregiver partners. Recruitment was conducted primarily using e‐mail and social media, and thus we may have reached individuals who were more technologically savvy and more integrated within Canada's patient, family and caregiver partner community; this may have resulted in a slightly skewed set of perspectives on virtual engagement and engagement during the COVID‐19 era. Further, the focus of this survey was not on COVID‐19 itself and individuals may have had more nuanced opinions on the impact of COVID‐19 if they had been asked additional questions on this specific topic. Finally, it is important to note that this survey was conducted in the Fall of 2020. In Canada, this was the beginning of the second wave of the pandemic. Perceptions may have evolved from earlier in the pandemic or later during subsequent waves.

## 5 CONCLUSION

This study provides a snapshot of patient, family and caregiver partners' perspectives on the impact that the first and second waves of COVID‐19 had on their engagement activities. As we begin to consider the post‐COVID‐19 era, identifying what has, and has not, worked during this time of intense difficulty is important. These lessons can help to create engagement programmes that build on successful adaptations and innovations that address many of the challenges faced during the pandemic. If patient, family and caregiver engagement is to be fully integrated into the health system, it cannot stop during times of crisis. Reflecting on how patient partners were engaged and not engaged) during this time offers constructive lessons for sustaining patient engagement. While many struggled with virtual engagement, the impact of these struggles was amplified by the COVID‐19 situation more broadly (e.g., social isolation, increased pressures on employees, caregivers and parents, etc.). Understanding the benefits and challenges of virtual engagement opportunities moving forward will also be helpful as health system organizations look to engage a more diverse set of patient, family and caregiver partners.

## CONFLICT OF INTERESTS

The authors declare that they have no competing interests.

## AUTHOR CONTRIBUTIONS

All authors contributed to the study conception and design, including the development of the survey tool and participant recruitment. Data collection and analysis were performed by Laura Tripp, Jeonghwa You and Julia Abelson. The first draft of the manuscript was written by Laura Tripp and Julia Abelson. All authors critically revised the manuscript and have approved the final version. Julia Abelson and Meredith Vanstone are the Co‐Principal Investigators of the Canadian Patient Partner Study.

## Data Availability

The data that support the findings of this study are available from the corresponding author upon reasonable request.
